# Supplementary material for: Patterns of Intron Gain and Loss in Fungi
Source: PLoS Biol. 2004 Nov 30;2(12):e422. doi: 10.1371/journal.pbio.0020422 (PMC532390; doi:10.1371/journal.pbio.0020422)
Supplement: Table S1 — Also available at http://genes.mit.edu/NielsenEtAl/. (4.3 MB ZIP). [file pbio.0020422.st001.zip › NielsenEtAl/html/808.html]

AN7303.1.NCU00164.1.MG05687.1.FG02527.1


```
 CLUSTAL W (1.82) Multiple Sequence Alignments - Introns Inserted


Sequence 1: MG05687.1	1052 aa
Sequence 2: FG02527.1	991 aa
Sequence 3: NCU00164.1	1043 aa
Sequence 4: AN7303.1	950 aa
Alignment Length: 1111 aa
Number Identitical Residues: 317 aa
Alignment Score (without introns) 18334


MG05687.1 	M0VLFKRKPVRFLEPPQVPDGSSE0VWYIDQT~GEMFLTYEDYLNR~MDFYK-~-----Q
NCU00164.1	M0VLFKRKPVQFLTVPNIEDDNQE0VWHIPQT~GEIFVTYEEYLNR2MDFYN-~------
FG02527.1 	M0VLFKRKPVRFLPAAEIEDENAE0VWHIPQT~GEVFTSYDSYLDR2MDFYKQ0RRFNDQ
AN7303.1  	M0VLFKRKPVQYLPRPIIEDDSSE~ACTLRED1-------------~------~------
          	* ********::*  . : * . * .  : :                             

MG05687.1 	0ITGHSGLTFFDALKSE0LHGAQEVDQAFPESLKGPILRRVQFSTVSRIDHLV~DQVFD~
NCU00164.1	~---------------Q0TAGAAEVEQAFPEALKGPVLRRVQFQTVSRIDTLV~DQIYD~
FG02527.1 	~ITGHSGLTFFEALKSE0LAGGKEVEASFPEALKGPILRKVQFQTVSRLDNLV~DQIYD~
AN7303.1  	~----------------~-GGIKRGEQLVSRSAKG-----SHIAPGPILD--V1DEIYE0
          	                    *  . :  ...: **      :: . . :*  * *:::: 

MG05687.1 	EFKSDYYPGESVGIQLMEGDRHQGVVREKVKFPSKMLPDGSS-TKPYSSYTVSLDSMPGQ
NCU00164.1	EFKNDYYPGETVTVSID-GEKLHGVVRDKTRFGGTVLPDGST-APPYSRYFVSLKERQ-E
FG02527.1 	EFKHDYYPGEEVTVTMKGGDRAHGLVRDKTTFGPRALPDGSH-SLPTTRYLVDLKDSE-E
AN7303.1  	EFKSEFYPGEPVLILLDDNTRLHGVIRDKAKFPEQLYPDGTVRTPAHATYLVKVLDRPDE
          	*** ::**** * : :  . : :*::*:*. *     ***:  : . : * *.: .  .:

MG05687.1 	EAQVDSEHIWRDRKVFTKSVLRSFIKKTVTREAWNGAPWLVKHDVAAKYHIDTRVPAHLL
NCU00164.1	EVPVNDSQIFRDRKVFTKSVLRSFIKKTVTREAWNGAPWLVKTDIAERYHIDTRIPPHLR
FG02527.1 	ETIVTDEHICRDRGIFTKAMLRSFIKKTVTRDAWNGAPWLVKHDYASQYHIDTRVPPHLR
AN7303.1  	EALLDNHHITRDRKTFTKQMLRAFIKNNVTRESWNGAPWLVKPSVAEEYRIPTEVPKHLQ
          	*. : . :* ***  *** :**:***:.***::********* . * .*:* *.:* ** 

MG05687.1 	YDNKLLERKHLQAQKKHQN----STDSSFNGTNGTPPVNGNGTMPTNPFSSNGPMRLPEL
NCU00164.1	YDNKLLERKQIQAQKRMTQPGGMGMEFNSHTSHNTPASPGQ-------FSPTGPVRLPEL
FG02527.1 	YDTKVMERKQLQAQKRAHT----HHDT--NSHDLIPALQ------------SGPVRLPEL
AN7303.1  	YGARVAEKK---AQKKADQEGFFGFFASKELPELKPAVKGQ-------KAKPSAQDLARS
          	*. :: *:*   ***:    .      . .  .  *.  ..        :  ..  *.. 

MG05687.1 	KPAPKSHKPKHLQGQQPTKGKLTGLVHPEICHDMMPPPMPGNPLQFSMPYQNSGKTIPMM
NCU00164.1	KPAPKSHKSKAQQAAQAAAQEAR----------QNGGVVSRKHNLFNGPE--------PG
FG02527.1 	KPAPKSKAKPGQHGSGSKGLKWP----------LNMPVHGGNP--FSSPS-DYGIQAPAQ
AN7303.1  	EEAQFLEYKRSLNGNPSFLVGNK---------SMNGPPRPSKS---QEPEKNAPPIPSVV
          	: *   .     :.  .               .        :    . * ..     .  

MG05687.1 	Y~QPEPP-PPPPPP-KYPIEDLQLESRG---HVRPKIKFLCKDPPVDTEKTPPLG----E
NCU00164.1	G2APEPP-PPPPPP-KYPIEDLQVESRG---LVRPQLKYLCADPPVDLEPTFKAP--FDG
FG02527.1 	R~EPTPP-PPPPPPPKYPIEDLQLEPRDD--RKRPPLKFLCRDPPAKVENEDAIQDDFSD
AN7303.1  	I~KAEPPRPPSPPPIKYPIEDLEIPPNREKKKQRPPLKFLKANEFDDPNDEDLLQ----D
          	   . ** **.*** *******:: .. ...  ** :*:*  :   . :           

MG05687.1 	KILMKSVGPLLETWDTLNVYCEVFKLDSFTFDDYVETLVVTSEEVRPQLFDEIHCSILSV
NCU00164.1	KILMKSVGPLLETWDTLNVYCEIFKLDSFTFDDFVEAMQLASEEMPVQLFNEIHCAVLKI
FG02527.1 	NINMESIGPLLETWDTLNVYCEVFKLDSFTFDDYVEALCVASEKVPIQLIDEIHCSVLKI
AN7303.1  	AIEMESVGLLLETWNTLNVYCEVFQLDSFTFDDFLQAMRFSSDEMDCELFVEIHCAVLKK
          	 * *:*:* *****:*******:*:********::::: .:*:::  :*: ****::*. 

MG05687.1 	LVNSEKD-GGEAQIQLPALDD-DEDDDEE-----EEESRSPTPEPEPKPAKRATRSSLAK
NCU00164.1	LVSSEAD-GGKVQIQLPELEE-EDEEEED-----EEDSAAATPEPESLPTARATRSSLAK
FG02527.1 	LVDSEAD-GGKVRINLPEVEEEDSEEEED-----EDASTLPTPEPEVKPGRRATRSSLAK
AN7303.1  	LVNAEKDDNGAIQISLPELPVEESEDSDEDEEMVEDEQDEPEATPEPAVTRMTTRGSLAK
          	**.:* *..*  :*.** :  .:.::.::...  *: .  . . **      :**.****

MG05687.1 	AEAERLANEAAAAERESEEQAALAAKRHRAEDVLEDYDWVEQAAKRNFQDGGWERIMVGL
NCU00164.1	LEAERLAAEAAAAEREMQEAED--APKHRAEEVLKDYDWIEHLRKRDFKDGGWELIMVGL
FG02527.1 	AEAERLAAEAAAAEKESEE-----ETKHRAEQLLEDYDWIEHLRKRDFSNGGWERILVGL
AN7303.1  	AEAEGLKATLNGDHDSSE------IKSHRASEMLSEYRWIDRLRKRDFRNGGWEMIMIGL
          	 *** *     . . . :         ***.::*.:* *:::  **:* :**** *::**

MG05687.1 	LYQISQRERLHDTCEDILAQLVPTA-DHPTQETVRQNYATMDVNMRVDALQLICMLTVET
NCU00164.1	LHQLSKNERLNASCEELLEQLVPVEDEEPSRETVRARYASLDVNYRVQALQIICMLTAET
FG02527.1 	LHQLSENERQHDICEELLHLLVPAG-VDATQETVKQRYAELDVNQRVKILQIICMLTTET
AN7303.1  	LHQLSVRPRMEKVCNQILEHLAPLD-QEPTQDTARAQYAVLDVNLRIRALQIICMLSLET
          	*:*:* . * .  *:::*  *.*    ..:::*.: .** :*** *:  **:****: **

MG05687.1 	KAVRAYMEECSETMTEYRKQKIELQRQRKLA2IEELKALNEERKIQLPDN-MPPSPKPEV
NCU00164.1	KAIRGYMEDCSEQMTAYRKEKIEWQRKRKQA2LEDLKNLNDQRKILLPDN-MPPSPPLES
FG02527.1 	KAMRGYMEDCSETMTAYRKERIEWQRQRKQA2IEDLRQLNEQKKILLPDN-MPPSPPKEE
AN7303.1  	RAIRNYLEECSNQMTEFRKEKIEYQKARKAA2MEEVRRLHQECKALAPEPEKSPSPLPEM
          	:*:* *:*:**: ** :**::** *: ** * :*::: *::: *   *: . .***  * 

MG05687.1 	PKS--------NGDVSMTGTNDHASP---LVDDDTMNVDTTEDDVAIGRGLRRAQDRAAQ
NCU00164.1	AKVNGVNGAHPNGDVKMTDVDEISLTGGVHISDEIPDSDAEDADETPTNGLRNLR-RGND
FG02527.1 	-----------DGDVNMAEPDDSIMS---READG----EETEEDPSTRKRRRPLTAKQRK
AN7303.1  	EP---------AEDTKMSGMDG----------------DSQVESEVEGTPQRALR-GGVD
          	             *..*:  :                 :    .       *       .

MG05687.1 	RQKKRELEEERKREKEAAAAT--KAPKQTKQFIKLLKDIQKKEDTIKELEAEIAVIDNDL
NCU00164.1	RAAERKRKEEEKKQRAEAAAAAAKVPKQSKQFLKVLKDIQKKEDEIAECEKEIAIIDNDL
FG02527.1 	QEEEQALKKEKEKEKAAKMAKTPKPPAQSKQFIKLLKDIQKKEDVMKKCEEEIAVIENDL
AN7303.1  	RVMERKRKLEEERERKEQLAK---QPKGSKQYQRLLKKLEEQKAAVKKFEDKIAVVDNDL
          	:  ::  : *.::::    *     *  :**: ::**.:::::  : : * :**:::***

MG05687.1 	READCPRTRVLGKDRFWNRYYWFERNGMPYAGLPQSSTAEAEYANGCIWVQGPDDMEREG
NCU00164.1	READCPRTRVLGKDRFWNRYYWFERNGMPYGGLPDSSTAHAGYANGCIWVQGPDELEREG
FG02527.1 	READCPRTRVLGKDRFWNRYYWFERNGMPYGGLPDSSTASADYANGLIWVQGPDELEREG
AN7303.1  	READCPRTRCLGKDRFCNRYWWFERNAMPYEGMPDSSTAEAKYANGRLWVQGPDDMERLG
          	********* ****** ***:*****.*** *:*:**** * **** :******::** *

MG05687.1 	YIDLAPEQEKEYKAKFGMTVAERKVKDEGPTSVFNARQWGFISDPDDVKKLIEWLDPRGF
NCU00164.1	YIDMKEEWQNEYVAKFGITVPERKKREEGGTSVFKATQWGFYERAEDVDKLLEWLDPRGV
FG02527.1 	YIDLPADLQEEYKAKFNMTVPERKAMEESNTSVFNAHQWGYISEPEHVDELIKWLDPRGF
AN7303.1  	FIDVSDDLKKQYQKRFHMSPAERKKHEEGPTRLSGAHEWGYYDEPEAIEKLLDWLDSRGE
          	:**:  : :::*  :* :: .***  :*. * :  * :**: . .: :.:*:.***.** 

MG05687.1 	NELKLRKELVAFKERIETHMRNRLKYIGNP--EEDKPVEE-----------TDKKERTPE
NCU00164.1	NETKLRKELVNYRDRIQKGMENRKTYVGLDTATEEEKKEENGATGGDAMEGVEASTTAAV
FG02527.1 	NELKLRKELLAFKEKIAKHMQSRKTYLSAE--EENDKKEE-----------TQKR-----
AN7303.1  	RETKLRKELLLHRDKLVKCMQARHEYLSRT----EDDSED----------IPTKR-----
          	.* ******: .:::: . *. *  *:.      :.  *:                    

MG05687.1 	KEPERKSGFTAVNSGKRSTRGG--VAAKVAVVKSTPEPPQYRCLKWENQTMIEDEGHIHS
NCU00164.1	VEEQPVQPTTKGGRGKRSTRGNNNSRAATTTRQVTPEPPAYRCLAWVNTTAIEEIGHLHG
FG02527.1 	----------------MSTR----------IRDKTPDTSNYRSLQWENTTALEELGHLHS
AN7303.1  	----------------VTTR-----------NKTYVADAKHRCLNWRNSTALADNGHLHV
          	                 :**            .     . :*.* * * * : : **:* 

MG05687.1 	VHPPPPRTKKPSRKK-EAADASAAAEKKGKSLGRQGTRYNF-
NCU00164.1	EEPPPARNRKQSKKKQEAAAAKEEVEVQVAPTTRSGKKKSAR
FG02527.1 	DPPPPPRSRKQTRKR-EAVNDAPAPAPAAKTRRR--------
AN7303.1  	DASRPS---KRARKSGEDLKEVKATNRQGKPLTRQGGRYNF-
          	  . *.   * ::*  *             .  *..   .
```
